# Investigation of distribution system modification using operation voltage unification in order to decrease interruptions in dust climate

**DOI:** 10.1016/j.heliyon.2024.e37335

**Published:** 2024-09-02

**Authors:** Hadi Norouzi, Mahdi Golchoob Firoozjaee, Majid Rezaei

**Affiliations:** Niroo Research Institute, Tehran, Iran

**Keywords:** Ahvaz, Distribution system, Decreasing, Fine dust, Medium voltage level, Failure, Insulation, Unification

## Abstract

Many events in power networks which caused failure and outage of subsystems have root in design and initial selection of equipment and their specification. This concern will be important while the any kind of expanding and acclimation plan need to be executed to strengthen the existing infrastructures in order to provide better condition with regard to satisfied criteria. In Iran one of the most adverse environmental occurrences known Fine Dust, may force several distribution utilities to be changed due to new technical requirements. Medium voltage level regrading in way to decrease or unification of distribution system have been proposed in this paper to reach reduced faults and fast replacement of equipment in damaged situations. Voltage level changing and selection have been investigated in order to increase the network strengthen and reselection of equipment and redesigning critical sections of system against natural events that called fine dust with aim to attain an upgraded distribution utility. Operating of existing 33 kV and 11 kV network in 20 kV level and investigation of changing process have been done for Ahvaz medium voltage distribution system as a case study in these paper's sections. Calculations and results will be contained maximum current capacities in different line conductors, insulation and air clearances assessment, poles acceptable mechanical values, short circuit current and protection coordination for changing voltage level in distribution system.

## Highlights

• Each development plan can be prepared to improve functions and equipment in order to solve system challenges.
• The Ahvaz distribution network consists of two sections, the west and the east, which have voltage levels of 33 and 11 kV.
• By reducing the voltage level from 33kV to 20kV line current increase and thermal challenge will be shown in this change.
• In the case where the line voltage decrease, there is no need to change and increase the clearances.
• Voltage decreasing from 33kV to 20kV be necessary uprating the existing conductor to new higher cross section and weight.

## 1 Introduction

The modification and alteration of the distribution network is one of the fundamental attitudes of the energy distribution utilities, which has been taken into account concurrently with the construction and expansion of these networks. Each development plan can be prepared to improve functions and equipment in order to solve system challenges such as loading growth, financial resource reduction, reliability interruptions, losses, exhaustion of equipment and processes, progress trends, climate changes, adaptation of regulation rules to new conditions, monopoly prevention, specification unification, and other technical issues [1].

One of the most important factors that cause faults, failures, and outages in the power system are events, which, as an external item, can create various mechanical, electrical, and thermal stresses on equipment and disrupt the normal operation of the network as a result. Natural events such as lightning, storm, wind, snow and ice, drought, sandstorms, temperature increase and decrease, humidity changes, earthquake, and flood cause in the system in terms of the type of fail, equipment damage, duration and stability of the disruption in the network, the degree of impact on network security and other things, are different from each other. So investigation of these events effects on distribution systems needs determination and analysis of aspects and features as complete as possible.

Literature review of this paper have two main contains that one of them is improvement of system operation, reliability and resiliency against natural events and other is redesigning of distribution system. According to contribution of this paper which is the investigation of system modification in fine dust contamination by using operational voltage changing many articles, reports and standard sources have been used as references.

In [2] the resiliency of power network and Extreme weather hazards impact on critical infrastructures have been investigated as a framework for operation and restoration of power system which adapted to grid's situation like to be smart and uncertainly. In Ref. [3] active distribution systems have been focused as a vital part of energy systems and a framework has been proposed for conventional distribution systems for smarting as resilience investigation against natural disasters. In Ref. [4] under budgetary constraints and based on a prioritization scheme which have been formulated to inform modified strategies to strengthen grid resilience. As a case study of the distribution system in Connecticut, the model was trained on historical outage data from storms between 2005 and 2020, and a counterfactual investigation is analyzed. urban areas with smart grid technologies have been focused on enhancing resilience oriented in the electrical distribution grids in Ref. [5] and as a natural event subject, the pole outage by hurricanes in the electrical distribution grid is introduced to scenario generation via the pole fragility function. for enhancing resilience against events at day-ahead the short-term reserve scheduling is proposed as a pre-event response. In Ref. [6] the topics and the papers selected for the Special Issue on Flexible and Resilient Urban Energy Systems summarizes. 21 papers are accepted for publication After rigorous reviewing process.

The resilience of power distribution systems has been assumed that depends on two main distribution infrastructure and restoration priority factors in Ref. [[4], [7]] and modeled as outage duration in the residential electrical power distribution system in part of the City of Phoenix, Arizona between 2002 and 2005. To maximize the resilience of a distribution system and minimizing the total cost based on a customized genetic [[5], [8]] proposes a single-stage multi-criteria optimization model through a series of investments that included storage and photovoltaic units, and the undergrounding of overhead lines and the importance of managerial and logistic training in distribution companies have been mentioned in results. The objectives of paper in Ref. [[6], [9]] are the resiliency assessment and improvement of design criteria for an urban distribution system against during many winter storms that is used a combined statistical–geographic information systems approach. In Ref. [[7], [10]] the incorporation of technologies, such as micro- and smart grids has been a vital research field of planning and operation of electric power systems and make the distribution system resilience in the active distribution system (ADS) during, and after an extreme event by distinguishing characteristics between reliability and resiliency [[8], [11]]. Provide a probabilistic multi temporal and multiregional resilience assessment methodology, based on optimal power flow and sequential Monte Carlo simulation with worth index of the individual transmission components. The methodology by using a mix of infrastructure and operational indices is demonstrated using a test version of the Great Britain's system. For minimizing the adverse impacts of extreme weather events [[9], [12]] provides a mitigation strategies and analysis of existing research on planning solutions to enhance distribution system resilience and support power distribution system operators and planners [[10], [13]]. by integration of Battery energy storage system to proposal model make a comprehensive robust approach for the reliable and resilient distribution expansion planning, where the concept of multi-micro grid as a target solution is adopted for reducing the outage times.

The phenomenon known as fine dust is the most important climate and environmental change that causes many events and outages in several provinces power distribution systems in Iran, especially Khuzestan [[11], [14]]. The most significant climatic and environmental change that affects Iran's electricity distribution networks, particularly in Khuzestan and other regions, is the phenomena known as fine dust. Wide interruptions in the system caused by electrical insulation failures in fine dust climate are the major problems which need to be investigated due to the fact that there is many equipment in the power distribution systems in the open environment, so the composition of this contamination and other environmental conditions like rain, fog and humidity can reduce electrical withstand voltage. The majority of Iran's medium distribution networks operate at a voltage level of 20 kV, while other regions, like Khuzestan, have 11 and 33 kV. The operating voltage of the MV (medium voltage) network has a great effect on the structure and characteristics which determines the maximum length and number of each feeder, it's loading and distribution substation supplied, system losses, reliability, insulation and protection coordination, operation plans, control, repair and maintenance, and finally on annual costs and investment planning. The measures that have been taken for increasing system resiliency in fine dust pollution and condition are based on short-term, medium-term, and long-term methods such as washing insulators, using silicone coatings, or upgrading Equipment. Changing the voltage level from 33 to 20 kV which is presented and investigated technically in this paper can be a solution and alternative operating voltage of existing the Khuzestan network for reducing insulator faults in order to growth of withstand voltage and unification of equipment specifications with other distribution regions of the country.

As follows, various experiences for changing and modifications of distribution networks with a focus on MV, such as reducing the number of voltage levels from two to one, increasing the voltage level, and structural changes with the aim of strengthening and developing the existing network, are explained. In Ref. [[12], [15]] using a wide range of studies, possible alternatives for the Coventry distribution network, the urban areas in the British electricity network which is 0.4//6/6//33//132 kV, have been investigated. Part of the Coventry distribution network includes the 6.6 kV network, which according to the expansion of the distribution network in England, this part of the Coventry network is known as non-standard voltage level. In addition, the operators of this network are trying to eliminate the 6.6 kV network in their future design strategies for the development of the distribution network. Due to this, various design options have been considered to replace the worn-out 6.6 kV network. In these studies, the performance of the possible future network with one MV voltage level, such as the 132/0/11//132 kV and 0.4//20/132 kV networks, has been compared with the performance of the current design with two MV voltage levels. In this study, three different design options, including the number of voltage levels, the voltage value of each level and different network design criteria, have been taken into consideration. The potential techno-economic performance of the network is investigated by converting from the existing two MV voltage level design to a single MV voltage level design with the remove of 33 kV. In addition, the 20 kV system was selected as one of the design options to compare with the 11 kV level. Finally, the role of losses in the design of the network with the aim of providing peak load compared to the design of the network with the aim of optimal economic operation has been shown. The 30-10/110 kV of Finland medium voltage system changing to 110/20 kV since 1960 over the years have been investigated in Ref. [1] and show the medium pressure system has progressed from two voltage levels to having a standard voltage level. In this revision and revision, it has been proven that the plan and project has been very successful, so that it has benefited the investors from the economic point of view. The project to change the voltage of the southwest distribution network of Finland from 10 to 33 kV–20 kV was considered a suitable option for the development of the distribution network. In this regard, the 33 kV lines in the new network were used, while the 10 kV lines in the new network could not be used and were removed from the circuit. Replacing 10 to 0.4 kV transformers with 20 to 0.4 kV transformers was a very challenging task because the blackout time of consumers had to be minimized. In the last stage, the underground cable network should be updated in urban and island areas. Finally, with the implementation of this project, its cost-effectiveness was proven and electricity with better reliability was provided to consumers. IN Ref. [[13], [16]] reinforcement of electricity distribution network by using and construction of a new 132 kV wood pole line and the installation of a 132/33 kV transformer have been done. A project which has been titled expanding and strengthening the medium voltage distribution network in Kosovo investigated supply, installation, testing and commissioning of 20 kV medium voltage in all areas that included the construction of new, double and cable lines and operation of various components of the network [[14], [17]]. The 10 kV voltage in the medium distribution network of Zhengzhou as the capital of Henan Province have been changed in the expanding plan to the 20 kV voltage level in 2010 and have been built a 20 kV test network [[15], [18]]. The standard distribution voltage of 20 kV was introduced to replace with the 15 kV network in for Phnom Penh which its operation of the main network was very low after a long period [[16], [19]]. With the introduction of GIS insulation equipment, the Turkish Electricity Company, after long-term economic studies [[17], [20]] made a decision that the voltage level of 34.5 kV should be replaced with 10.5 kV in this country.

In this paper two following of voltage changing conditions have been considered for Ahvaz network:

1 Operating of existing 33 kV network in 20 kV:

In this case, due to the fact that the existing insulation distances have been designed for the 33 kV as a result of reducing the voltage level to 20 kV, will have two major resiliency advantages:

- Decreasing of faults

Considering that the nominal voltage of the network has rated down, as a result events which caused electrical failure decrease so reliability of power distribution system will be improved.

- Fast replacement of damaged equipment and decreasing system outage time

Unification of Khuzestan distribution equipment characteristics with the other whole country will be a bless situation in fine dust contamination which lets utility to have a fast replacing of important outage elements and as a result, safe recovery.

2 Upgrading the voltage level from 11 kV to 20 kV

In general, increasing the voltage level in the system and as a consequent reinforcement of network has two important advantages that first is increasing the distribution line rating and capacity and the second is the same solving resiliency issues for reducing the voltage level to 20 kV.

In fine-dust contamination for 33 or 11 kV distribution voltage many equipment may been damaged or cause some failures and outage with regard to their inappropriate operation so it will need to replace and change with new that require enough quantity assets in network utilities which presently contain 20 kV as mainly. In order to explained fact unifying system with 20 kV voltage let to have fast replacement or connection to other sections of country to have reliable restoration that means to have an improvement of Ahvaz distribution system resiliency. In Iran, almost the whole distribution system has 20 kV voltage level except for Ahvaz, so transition to a 20 kV will be a national consistent and adjustment with basic and main network. The challenges and constraints of changes in medium voltage levels of Ahvaz distribution network will be investigated in this paper. 5 main section have been made this paper structure as 1- introduction which was expressed, 2- Medium voltage level change investigation and proposed method, 3- Case study, 4- Results and 5-conclosion that will express in next items.

## 2 Medium voltage level change investigation and proposed method

The changes of voltage levels in the distribution network have many challenges and limitations such as network load supply, voltage variation of feeders, line conductor thermal capacity, insulation levels, reducing losses, adapting to environmental that these issues will have been investigated in this section.

### 2.1 Thermal capacity of line conductors

By reducing the voltage level from 33 kV to 20 kV, line current increase, and maybe thermal challenge will be shown in this change. The thermal capacity of the transmission line depends on the following factors:

- The maximum allowable temperature of the conductor (T_C_)
- Environmental characteristics, including ambient temperature, wind speed, etc.
- The amount of convection(q_c_) and radiation(q_r_) heat loss rate
- The amount of heat absorbed from the sun's radiation (qs)
- AC resistance of conductor at desired temperature(R_T_)

Also conductor temperatures are a function of:

a) Conductor material properties (primarily electrical conductivity)
b) Conductor diameter
c) Conductor surface condition (primarily emissivity and absorptivity)
d) Weather conditions (air temperature, solar heating, wind speed and direction)
e) Conductor electrical current steady-state conductor temperature for a given current and ambient temperature.

To calculate the thermal capacity of the steady-state in a conservative situation, the worst environmental conditions and the maximum temperature of 75–150° depending on the type of line conductor are considered. The thermal balance equation in the steady-state for the conductor current will be as follows [[18], [21]]:

(1) $$q_{c}+q_{r}=q_{s}+I^{2}R\left(T_{c}\right)$$

So in order to estimate of the maximum line conductor current capacity (I) Eq. (2) [18] is used in calculation for the information which is given in Table 1.

(2) $$I=\sqrt{\frac{q_{c}+q_{r}-q_{s}}{R\left(T_{c}\right)}}$$

**Table 1:** Specification of medium voltage overhead line conductors.

Conductor name	Fox	Mink	Hyena	Lynx
Diameter(mm)	8.37	10.98	14.57	19.53
Surface area(mm2)	42.77	73.65	126.43	226.2
Weight(kg/km)	149	255	450	842
Tensile Strength (N)	12812	21312	39977	79800
Modulus of Elasticity(kg/mm2)	8100	8100	7700	8200
DC Resistance at 20 °C(ohm/km)	0.7822	0.7822	0.2712	0.1576

For ACSR conductors, the maximum temperature value is 80 degrees of Celsius. In this paper is assumed that there is no wind and only the natural convection around the conductor causes a wind of 0.61 m/s and the radiant power of the sun is 1023 W per square meter, the coefficient of absorption and reflection of the conductor is 0.5 [[18], [21]]. For some of maximum ambient temperature values, the maximum line conductor current capacities have been calculated and the results can be seen in Table 2.

**Table 2:** Maximum line conductor current capacities (Ampere).

maximum ambient temperature(Celsius)	Conductor name			
	Fox	Mink	Hyena	Lynx
40	175	246	341	483
45	162	228	315	446
50	148	208	287	405
55	144	186	256	359

It is need to be said explained that environmental factors affect directionally and non-directionally in thermal capacity of the lines which means a congestion for energy transmission that have been cleared in this section as a function with environmental inputs like ambient temperature, sun's radiation, wind speed. In Table 2 maximum ambient temperature assumed 40 °C until 55 °C in 5 °C steps and there is only the natural convection around the conductor causes a wind of 0.61 m/s and the radiant power of the sun is 1023 W per square meter. In fact, when distribution line carries the amount of power, its temperature increase compared to ambient temperature and the maximum current have been limited by maximum temperature of conductors which have been used in Eq. (1)and Eq. (2) as thermal balance equations. Also the insulation requirement and challenges for voltage upgrading in generally caused the air clearances modification as explained in section 2.2 and as specificity in Ahvaz medium voltage the electrical strength of the existence equipment have been designed higher than new voltage so there is no need to change the distances.

### 2.2 Insulation investigations

One of the most important acts that should be done in order to change of line voltage is assessment of the air clearances that have been explained in follows.

- clearances to ground, and other supporting and adjacent
- conductor phase to phase clearance
- clearance between earth wires and conductors
- clearance for live line maintenance

In the case where the line voltage decrease from 33 kV to 20 kV, due to the fact that the electrical strength of the existence equipment has been designed higher than new voltage so there is no need to change and increase the distances. In fine dust situation the critical leakage current which can be determined in Eq. (3) [19], will be reduced with regard to decrease of line to line voltage (VLL) in constant Creepage distance (Dc. Therefore, the probability of failure decreases that means increasing of the system reliability.

(3) $$I_{c}={\left(Dc/\left(V_{LL}\times 15.3\right)\right)}^{2}$$

Based on Eq. (3), for two equal contamination situations the reduction critical leakage current cause reduction of failure probability and interruption duration. In fact, the aim of this paper is investigation of voltage changing with target to reduce the faults caused by fine-dust, so improvement outage time will be a naturally expected consequence of be smaller of failures and damages amount.

In voltage uprating from 11 kV to 20 kV, different and various techniques which used to increase clearance, as follows [[20], [22]]:

- Application of new surge arresters (also in the case of replacing the 33 kV network voltage with 20 kV, if the arresters are not replaced, in the event of a transient overvoltage may residual voltage is created in the network)
- Re-tensioning the existing conductors
- Performing sag adjustments
- Increasing the conductor height at the attachment support (converting suspension strings to pseudo dead -end string)
- Increasing the attachment support height
- Raising and/or moving towers
- Inserting additional towers
- Performing terrain contouring in rural areas
- Different line compaction techniques
- Reducing distance between phase conductors
- Increasing distance between sub conductors in a bundle
- Converting low voltage double circuit line to high voltage single circuit line
- Use of polymeric insulators
- Cross-arm modification

**Fig. 1:**
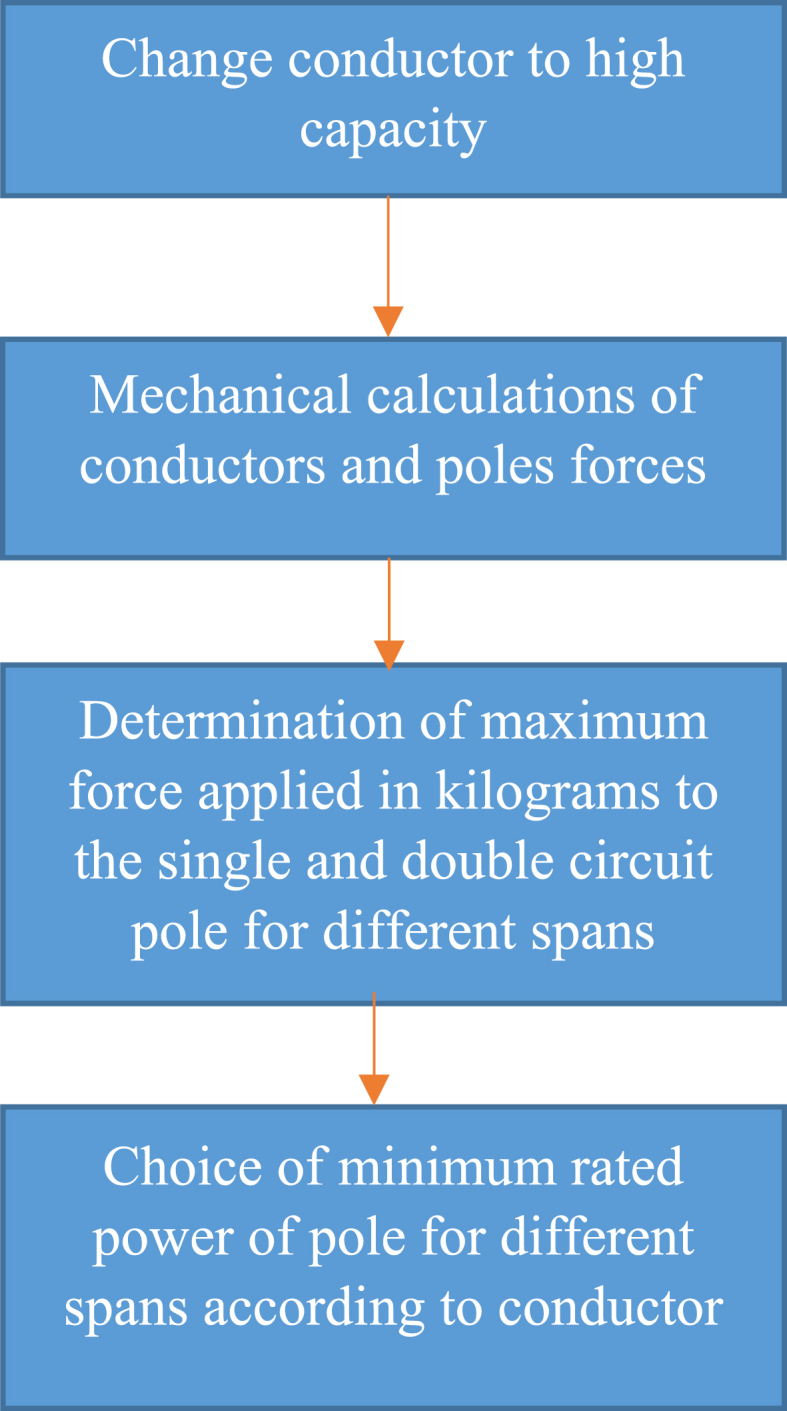
Methodology of selection acceptable minimum rated power of pole for changing voltage level in distribution systems.

Fine dust is a component of environmental contamination and weather parameters which including moisture and cause an anti-hydrophobicity specification for insulators that prepared leakage current increasing and failure probability as result so on of the best solution for this problem can be changing of voltage levels as proposed in this paper. In the medium voltage distribution network of Ahvaz city, considering that most of the 11 kV lines are designed based on the 33 kV, therefore, changing the voltage level from 11 kV to 20 kV is not costly and there will be no insulation challenges associated with increasing the voltage level. Also in order to basic design of Ahvaz distribution system based on the 33 kV, it is need just resizing of thermal conductors and mechanical pols without change in the spacing pattern of conductors (see Fig. 1).

### 2.3 Mechanical investigations

**Fig. 2:**
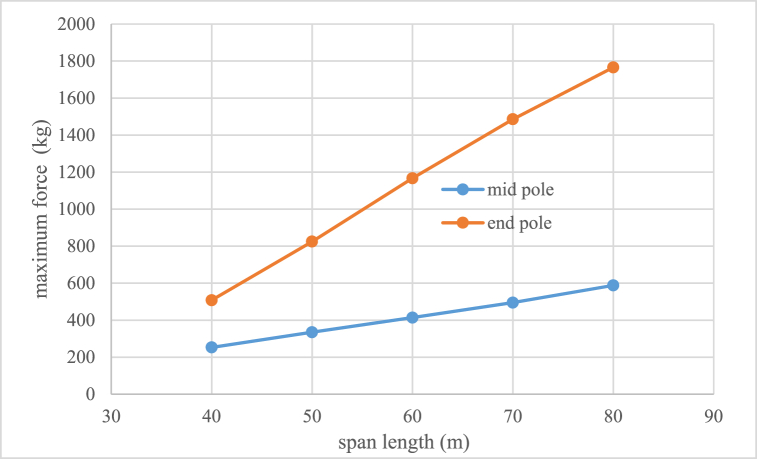
Maximum force applied in kilograms to the single Fox conductor circuit mid and end pole for different spans.

**Fig. 3:**
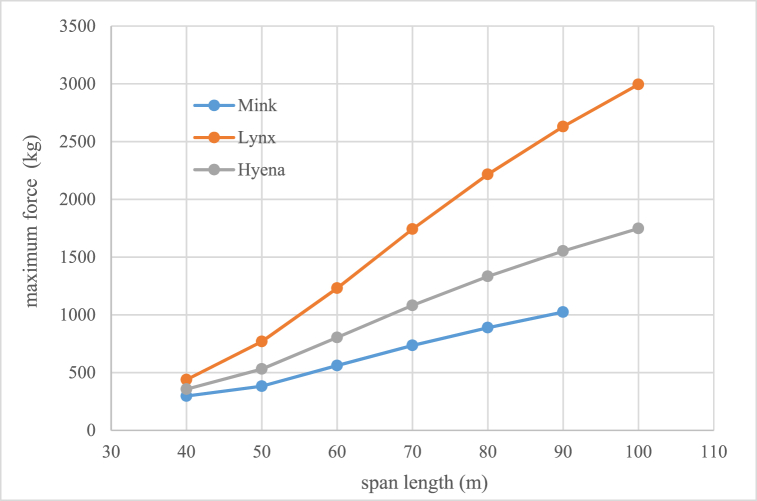
Maximum force applied in kilograms to the single type of conductor circuit mid pole for different spans.

**Fig. 4:**
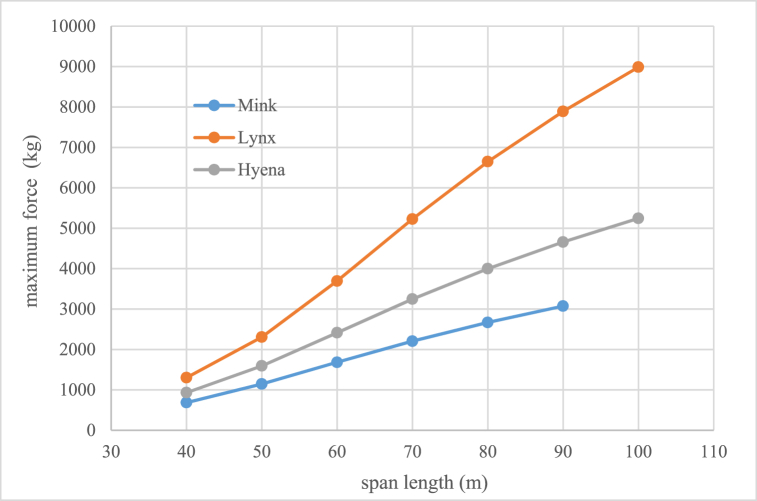
Maximum force applied in kilograms to the single type of conductor circuit end pole for different spans.

Voltage decreasing from 33 kV to 20 kV can cause lines congestion if load current would be over than conductor capacity then it is necessary to replace and uprating the existing conductor to new which has higher cross section and weight. Therefore, one of the existing challenges is investigation of mechanical ability of distribution poles which their information is given in Table 3. To withstand the forces that enter the conductor in different weather and system situations. The methodology of selection and choice of acceptable minimum rated power of pole is shown in Fiq.1 and calculation values for different type of conductors are given in Fig. 2, Fig. 3, Fig. 4. The basic mechanical design and calculation have been explained in Ref. [[21], [23]].

**Table 3:** Poles specifications with 12(m) length used in medium distribution systems.

Rated power (kg)	200	400	600	800	1200
Weight(Kg)	950	1580	2000	2500	3200
Top Dimensions(cm)	15*10.5	22*19	25*19	31*23	40*24
Down Dimensions(cm)	39*22.5	46*31	55*37	61*41	70*42

### 2.4 Protection coordination investigations

Change in the short circuit level for the distribution system nods is one of the important consequence of voltage increasing or decreasing in network. Therefore, current protection settings have a not correct function and it is necessary to make an adjustment. In HV to MV substations short three-phase circuit current in MV side (I_u_) is determined in Eq. (4) [[24], [25]]which u, X_u_, S_b_, %uk_k_ and X_l_ are respectively MV side voltage, transformer impedance in ohm, rated transformer power, transformer impedance in per-unit and line impedance in fault location, so the short circuit current rate of two new and old MV levels (u, v) can be calculated in Eq. (5)(based on Eq. (4)).

(4) $$I_{u}=\frac{u/\sqrt{3}}{X_{u}+X_{l}}=\frac{u/\sqrt{3}\times S_{b}}{\left(\%uk_{u}\right)\times u^{2}+X_{l}}$$

(5) $$\frac{I_{u}}{I_{v}}=\frac{u}{v}\times \left(\frac{\left(\%uk_{v}\right)\times v^{2}+X_{l}}{\left(\%uk_{u}\right)\times u^{2}+X_{l}}\right)$$

**Fig. 5:**
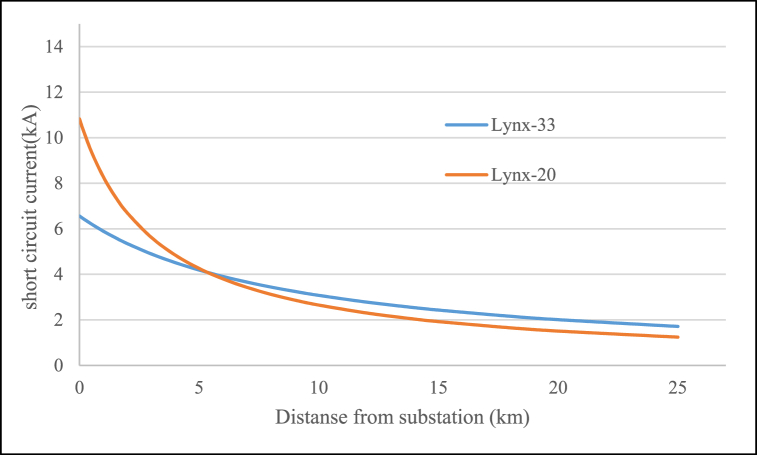
Short circuit current in two voltage levels for difference fault location distance from substation.

If nominal power of sub-transmission transformer be equal in two medium voltage level, it can be assumed also transformer impedance in per-unit will not be changed, so the ratio of short circuit currents in two cases has a reverse relationship with the MV voltage values. Decreasing and increasing Voltage from 33 kV to 11 kV–20 kV caused respectively increasing and decreasing of short circuit current in 1.65 and 0.55 times. For lynx conductor feeder short circuit current in two voltage levels are given in Fig. 5 in regard to difference fault location distance from substation. for MV to LV transformer and in Low Voltage (0.4 kV) side, short circuit current (I_l_) is determined in Eq. (6) [22] which S_tb_, %uk_kl_ are respectively rated distribution transformer power and transformer impedance in per-unit, so the short circuit current will have no change in LV network.

(6) $$I_{l}=\frac{{S_{t}}_{b}}{\left(\%uk_{ul}\right)\times 0.4\times \sqrt{3}}$$

**Fig. 6:**
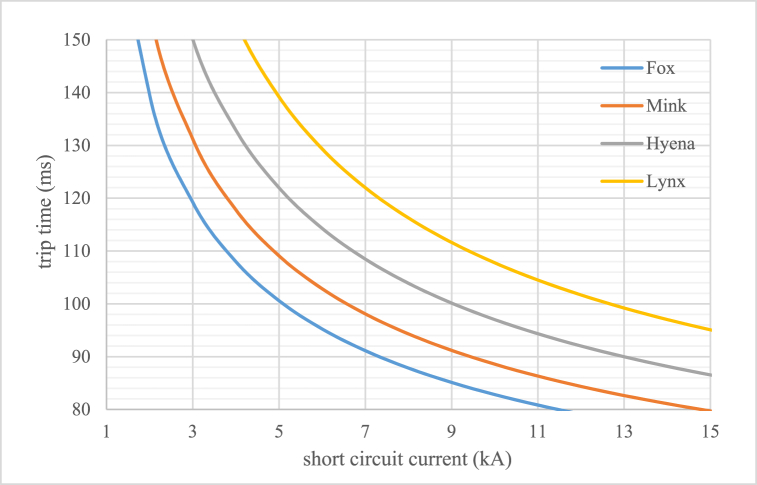
Reverse over current relay curves of type of distribution conductor.

Also according to operation time of protection devices such as relay are depending on initial setting and short circuit current, so in new voltage level with old input, trip time change and it is need to have another optimal coordination. In Eq. (7) and Fig. 6 [22] time and current setting and curves for reverse over current relay is shown with parameters as follows [[22], [26]]:

- t: trip time of relay
- I_b_: 1.2–1.3 times of maximum nominal current (current setting multiplier)
- I_ff_: short circuit (or input) current
- TSM: Time setting multiplier (that is 0.05 in this paper)

(7) $$t=\frac{0.14}{{\left(\frac{I_{ff}}{I_{b}}\right)}^{0.02}-1}\times TSM$$

### 2.5 Proposed approach and method

To compare the performance of the system before and after changes in the voltage level, it is necessary to make a proper evaluation of the system using some indexes such as total losses, voltage variation, percentage of line capacity loading, short circuit level and to determine the corrections approach and acts using the results of the calculations after approval new voltage level.

**Fig. 7:**
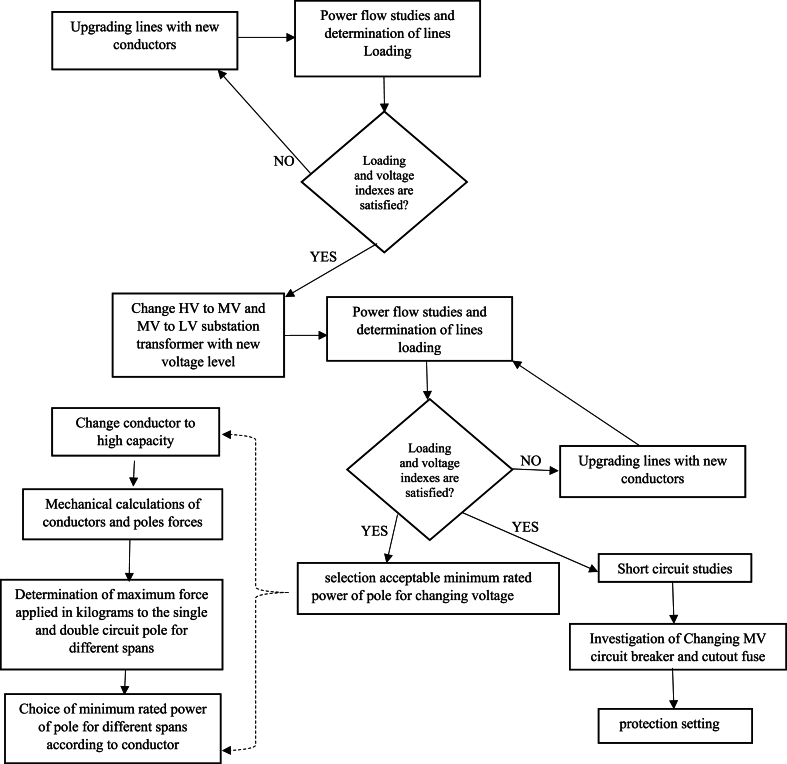
Flowchart of changing medium voltage of distribution system.

For example, increasing of loading caused many of lines will be overloaded or voltage drop in many nods, so these lines should be replaced with a higher cross-sectional area. According to explained items in this paper, Fig. 7 propose the series of methods and steps to have a distribution system with new medium voltage.

Loading and voltage indexes which have to be satisfied for a proper operation of changed system have been introduced as follows:

- Loading index: rate of conductor current to its ampacity in a feeder
- Minimum voltage: rate of minimum point in voltage profile to its nominal in percent.
- Maximum voltage: rate of maximum point in voltage profile to its nominal in percent.

Also one of the results in end of calculations is determination of main equipment which have to change such as arrestors, cut-out fuses, circuit breaker, line conductors, transformers and poles. In electrical and mechanical calculation, the environmental parameters have been consisted as input such as maximum thermal capacity of conductors or selection of poles power, so new equipment effects on system specification have been investigated such as coordination of protection relays and short circuits. that this proposal can be investigated for other case study with active elements as new experiment but the main result which have been attended base on pick of load will not change such as line capacity or mechanical calculations. It is noticed that one of the limits for proposal approach and proposal is network data. In fact, kind of data can make an issue for investigation of all constraints and studies that this expression can be cleared with regard to case study and result sections.

## 3 Case study

**Fig. 8:**
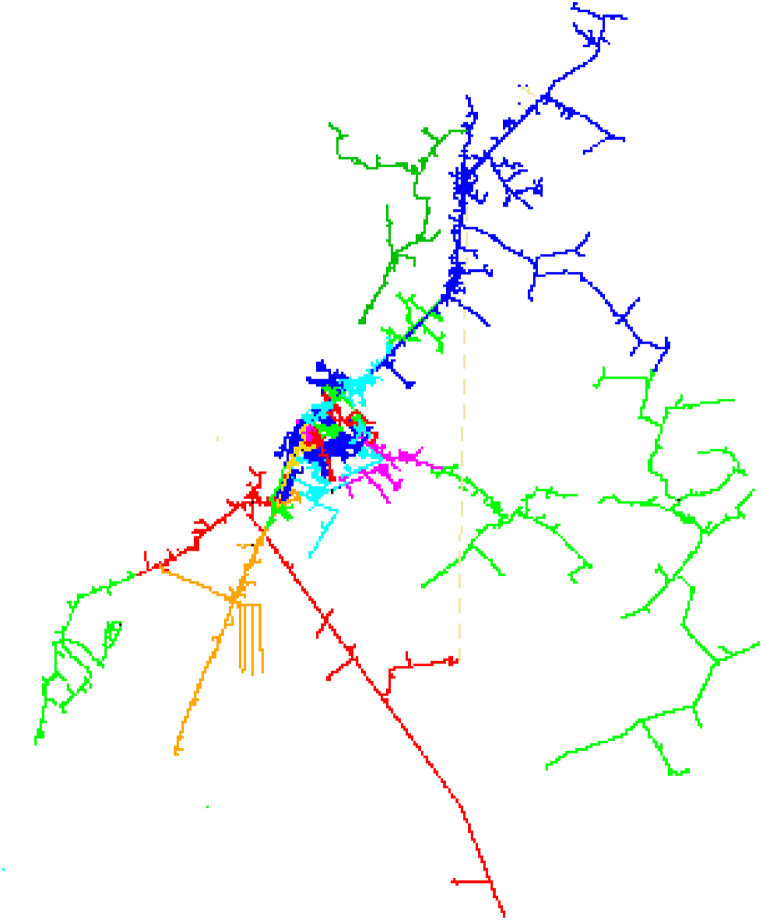
East Ahvaz Medium voltage distribution system.

**Fig. 9:**
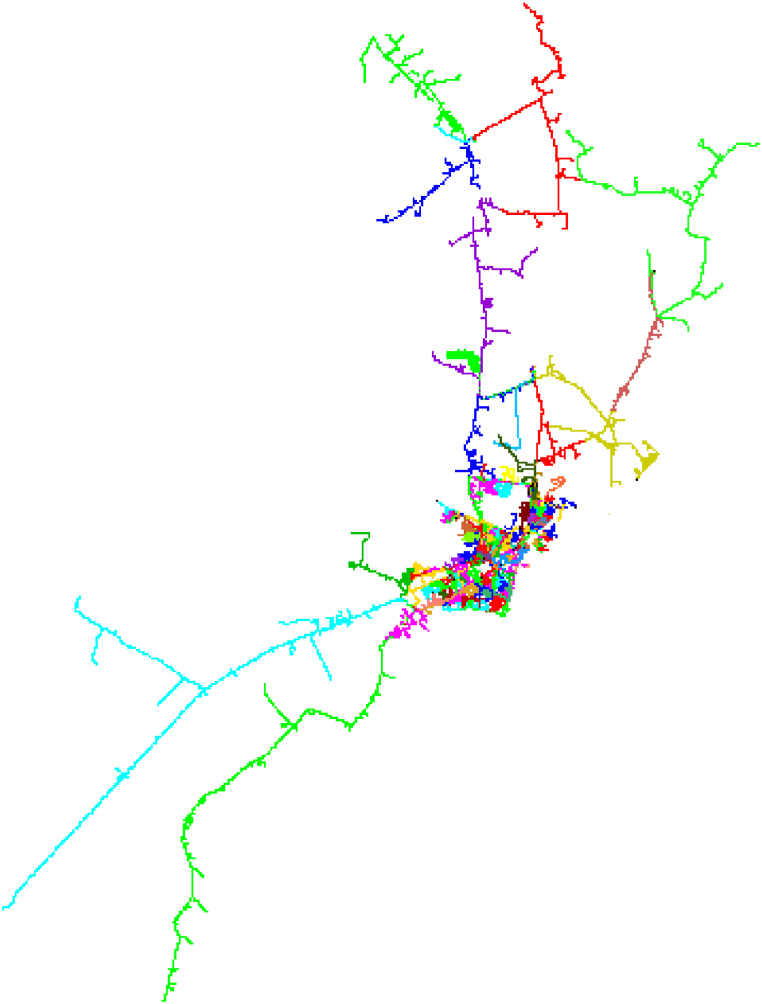
West Ahvaz Medium voltage distribution system.

The Ahvaz distribution network consists of two sections, the west and the east, which have voltage levels of 33 and 11 kV. The area covered by Ahvaz Distribution Company is the cities of Ahvaz, Hamidiyeh, Bavi (Malathani, Ramin) and Karun. The above sub-transmission substations have 132 to 11 and 33 kV transformers from which supply the 33 and 11 kV feeders. The distribution network of East and West Ahvaz are shown in Fig. 8, Fig. 9 for Digsilent software view in these figures, feeders differed from by colors.

The number of sub-transmission substations are 28 and 18 respectively in east and west sections.

The total of medium voltage feeders which come out of these HV substations are 137 and 28 in east and 92 and 39 in west section regard to 33 kV and 11 kV levels.

The total number of distribution transformers in East Ahvaz is 4987, and their number is as follows:

− 11 to 0.4 kV transformers: 377
− 33 to 0.4 kV transformers: 4610

According to the number of distribution transformers in each voltage level, it is clear that a high percentage of transformers are type 33 and only about 7.5 percent are type 11, according to this issue the main challenge regarding the replacement of transformers is related to the 33 kV network.

The total number of distribution transformers in West Ahvaz network is 6023, that 1202 and 4821 transformers have voltage levels of 11 and 33 kV, respectively. All the type of Ahvaz subtransmission and distribution transformers and conductors could be seen in Table 4, Table 5. For investigation of voltage change in this paper two distribution networks have been selected which supplied from Saheli and Golestan substation that their data are as follows:

**Table 4:** Ahvaz subtransmission and distribution transformers.

Rated power (MVA)	HV(kV)	LV(kV)	%uk
0.05-0.1-0.125-0.16-0.2	11	0.4	4
0.25-0.315-0.4-0.5-0.63-0.8-1-1.25-1.6	11	0.4	6
0.16	33	0.4	4
0.025-0.05-0.1-0.125-0.2-0.25-0.315-0.4-0.5-0.63-0.8-1-1.25-1.6-2	33	0.4	6
16	33	11	10
30	132	33	10.3
27	132	33	8–13
27	132	11	8–13
50	132	33	11–12
50	230	33,11	10–13

**Table 5:** Specification of lines conductors in Ahvaz distribution system.

Name	Rated current(kA)	Resistance(ohm/km)	Reactance(ohm/km)
Fox	0.142	0.9753	0.385
Mink	0.199	0.5661	0.564
Hyena	0.276	0.3369	0.348
Lynx	0.388	0.1965	0.329
Linnet	0.414	0.1868	0.335
ABC33KV35	0.128	1.11	0.1793
ABC33KV70	0.128	0.568	0.1595
ABC33KV120	0.184	0.325	0.1467
ABC33KV150	0.21	0.265	0.142
16cu	0.105	1.4188	1.558
CU25	0.136	0.9298	1.317
50Cu	0.208	0.4695	0.2937
35Cu	0.169	0.6562	0.3052
35CU-trans	0.122	0.62657	0.3052
cu70	0.252	0.3449	0.2937
2X33KV70	0.20792	0.342	0.194
2X33KV50	0.17079	0.494	0.2039
2X33KV300	0.4702	0.0785	0.166

**Fig. 10:**
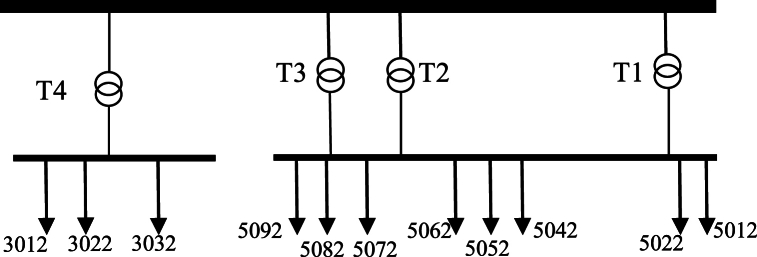
Saheli Network single diagram.

Saheli grid:

This grid is located in east and includes 4 transformers that three of them convert the 132 kV input to 33 kV and one of them to 11 kV which supply 8 feeders of 33 kV and 3 feeders of 11 kV. Single diagram of this network, transformers and data of feeders have been shown respectively in Fig. 10 and in Table 6, Table 7.

**Table 6:** Specifications and data of Saheli feeders.

Name	MV to LV transformer		Voltage (kV)	Total length(km)	loading	
	NO	Total kVA			Apparent Power(MVA)	Active power (MW)
saheli3012	16	6090	11	1.99	2.97	2.75
saheli3022	26	8240	11	4.98	4.11	3.79
saheli3032	30	9980	11	3.65	6.05	5.59
saheli5012	46	15180	33	6.89	8.19	7.6
saheli5022	31	9910	33	7.91	5.01	4.64
saheli5042	52	15485	33	11.01	6.43	5.95
saheli5052	6	1905	33	1.38	1.07	0.99
saheli5062	48	18005	33	9.12	9.26	8.59
saheli5072	27	8340	33	9.62	4.44	4.11
saheli5082	37	14825	33	4.88	8.04	7.47
saheli5092	62	24955	33	8.18	12.53	11.61

**Table 7:** Power transformer of Saheli Substation.

Name	Rated power(MVA)	Voltage		loading	
		High(kV)	Low(kV)	Apparent Power(MVA)	Active power (MW)
Saheli T1	30	132	33	17.1	16.61
Saheli T2	30	132	33	17.57	17.19
Saheli T3	30	132	33	17.57	17.19
Saheli T4	27	132	11	13.13	12.14

**Fig. 11:**
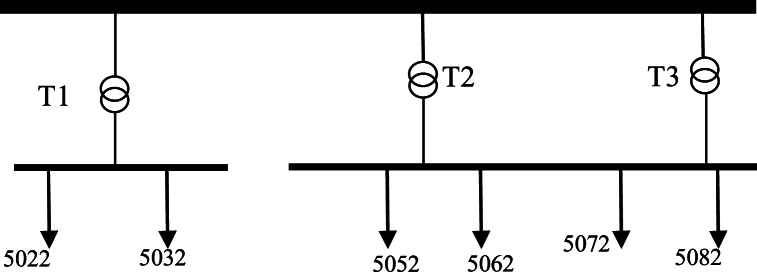
Golestan Network single diagram.

Golestan grid:

This grid is located in west and includes 3 transformers that convert the 132 kV input to 33 kV and supply 6 feeders of 33 kV. Single diagram of this network, transformers and data of feeders have been shown respectively in Fig. 11 and in Table 8, Table 9. Also there is a 33 kV to 11 kV transformer in one of the grid feeders that contain 11 kV distribution system with 12.3 km of total length of feeder.

**Table 8:** Specifications and data of Golestan feeders.

Name	MV to LV transformer		Voltage (kV)	Total length(km)	loading	
	NO	Total kVA			Apparent Power(MVA)	Active power (MW)
5022golestan	86	25290	33	11.73	13.5	12.5
5032golestan	69	24155	33	12.36	11.7	10.8
5052golestan	49	15055	33	4.69	7.1	6.6
5062golestan	4	1000	33	3.5	8.7	7.7
5072golestan	58	24355	33	5.18	10.6	9.8
5082golestan	58	21245	33	8.99	10.7	9.9
boostan3012	69	19795	11	12.3	8.05	7.32

**Table 9:** Power transformer of Golestan substation.

Name	Rated power(MVA)	Voltage		loading	
		High(kV)	Low(kV)	Apparent Power(MVA)	Active power (MW)
Golestan T1	27	132	33	24.18	22.83
Golestan T2	27	132	33	15.14	14.75
Golestan T3	27	132	33	21.3	19.75
Golestan2Boostan	16	33	11	8.06	7.35

## 4 Results

**Fig. 12:**
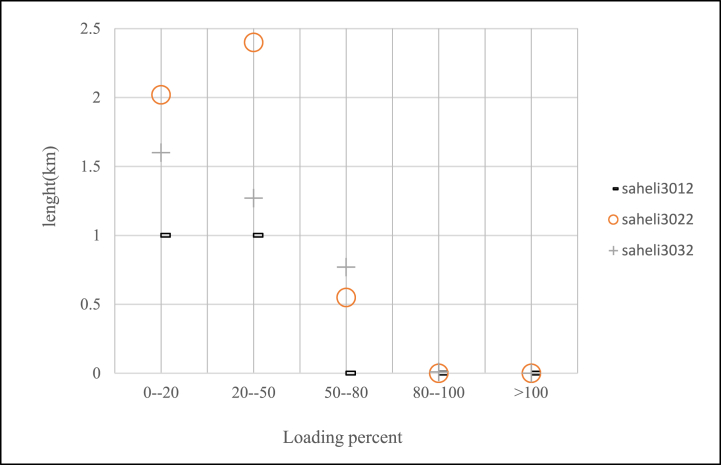
Saheli feeders loading after 11 kV–20 kV changing.

**Fig. 13:**
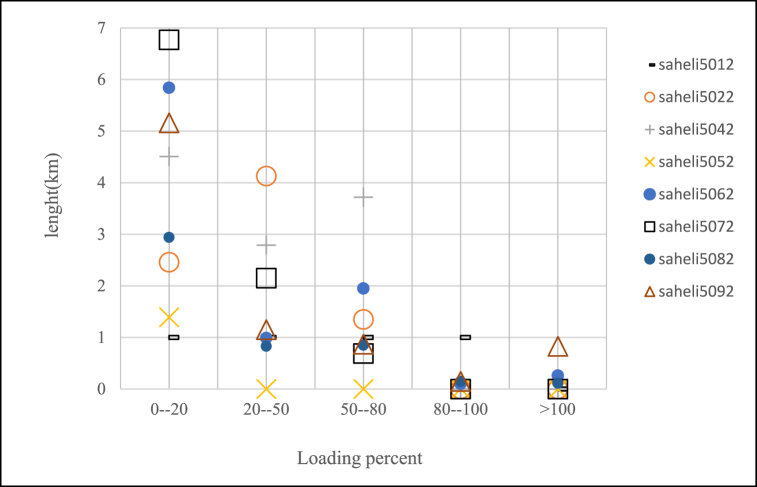
Saheli feeders loading after 33 kV–20 kV changing.

**Fig. 14:**
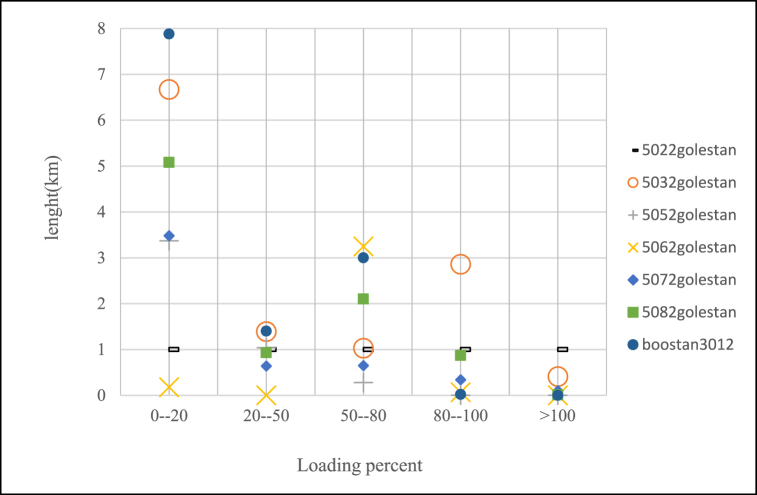
Golestan feeders loading after voltage level changing to 20 kV.

**Fig. 15:**
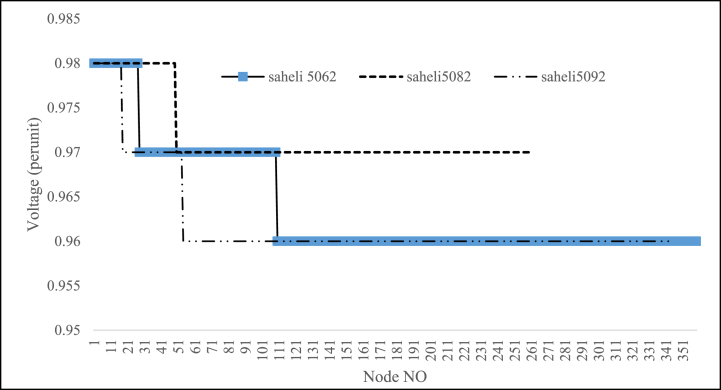
Saheli feeders Voltage Profile after voltage level changing to 20 kV.

**Fig. 16:**
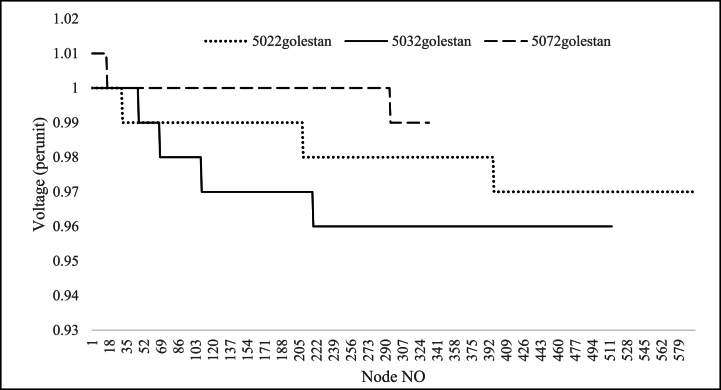
Golestan feeders Voltage Profile after voltage level changing to 20 kV.

According to case study which introduced in previous section, percent of lines loading for feeders after changing of voltage levels can be seen in Fig. 12, Fig. 13, Fig. 14. In this paper if loading percent would be over than 80 then some feeders need to upgrade of conductor as shown in Table 10. After voltage changing and lines upgrading done the voltage profile of feeders as criteria for this investigation have been exported and shown in Fig. 15, Fig. 16 which means a few lines have to replace of some loads to have appropriate voltage in constraint values.

**Table 10:** Feeders that need to upgrade the lines conductors.

Name	New conductor	Length(km)
saheli5062	Hyena	0.101
	Lynx	0.986
saheli5082	Hyena	0.113
	Lynx	0.148
saheli5092	Hyena	0.55
	Lynx	0.221
	Linnet	0.147
5022golestan	Mink	0.143
	Hyena	0.157
	Lynx	2.126
	Linnet	0.013
5032golestan	Lynx	0.408
	Linnet	2.863
5062golestan	Lynx	0.070
5072golestan	Lynx	0.440
5082golestan	Lynx	0.040
	Linnet	0.839
3022boostan	Linnet	0.018

**Fig. 17:**
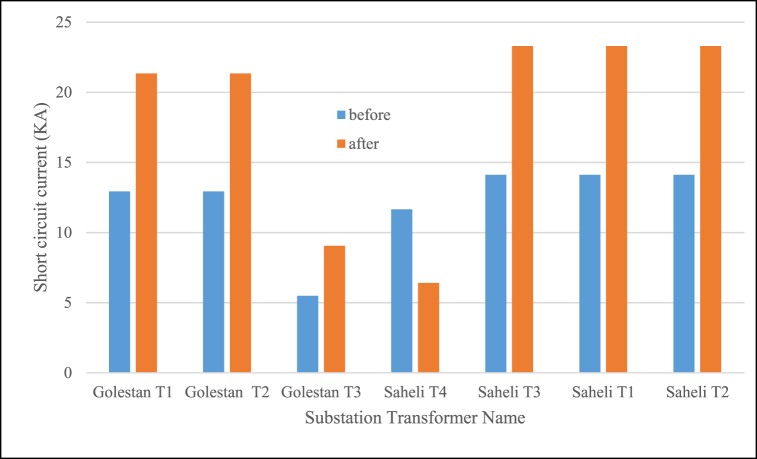
Short circuit current values.

**Fig. 18:**
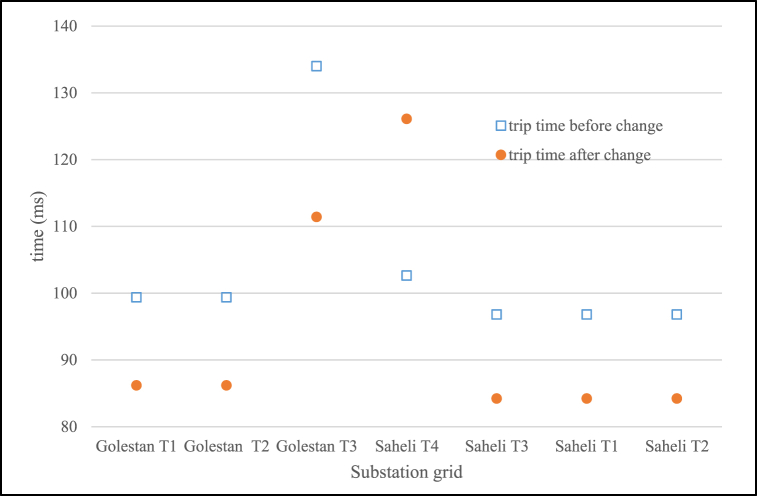
Main over current relay trip time in changing system to 20 kV.

**Fig. 19:**
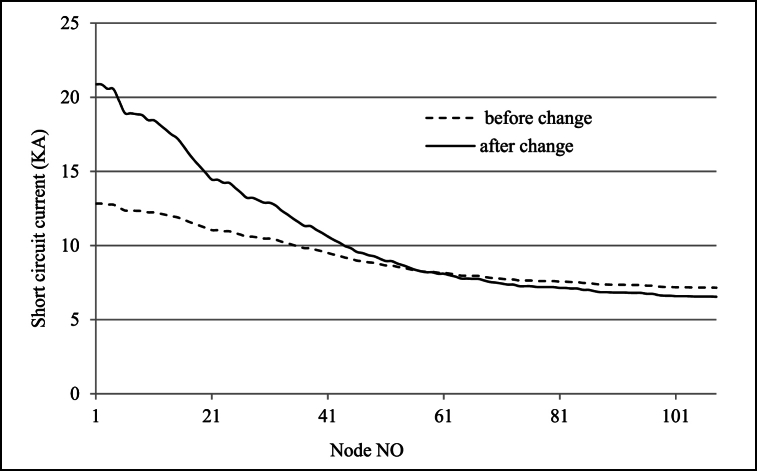
Short circuit current in two voltage levels for difference fault location distance from Golestan substation.

Also short circuit results while assumed fault have been located in MV side of substation are shown in Fig. 17, Fig. 18 for two case before and after Voltage level change. As expected for 33 kV–20 kV current values have been increased and in 11 kV–20 kV changing the values have been decreased. With regard to circuit breaker short circuit capacity (if assumed 16 kA in rms) the changing of equipment contains this disconnector element in distribution system for Golestan T1, T2 and Saheli T1, T2 and T3. Miscoordination of protection relays in order to new short circuit current for reverse over current curve can be calculated in millisecond as Fig. 14. The short circuit current in two voltage levels for difference fault location distance in one of the feeders from Golestan substation have been simulated and output is shown in Fig. 19 that to choice of cut-out fuses will be used. Finally, the investigation results of mechanical rated power values of distribution poles according to section 2.3 and Table 3 for different type of conductors are given in Table 11. In this paper have been contributed many technical investigation of system modification challenges that can be a solution for fine dust contamination interrupts, so reduction of failure and other advantages will be have cost benefits.

**Table 11:** Mechanical rated power (kg) values of distribution poles.

Span length(m)	conductor type							
	Fox		Mink		Hyena		Lynx	
	Mid	End	Mid	End	Mid	End	Mid	End
40	400	600	400	800	400	1200	600	2*800
50	400	1200	400	1200	600	2*800	800	2*1200
60	600	1200	600	2*800	800	2*1200	1200	3*1200
70	600	2*1200	800	2*1200	1200	3*1200	2*1200	5*1200
80	600	2*1200	1200	3*1200	120	3*1200	2*1200	6*1200
90	–	–	1200	3*1200	2*800	4*1200	3*1200	7*1200
100	–	–	–	–	2*800	5*1200	3*1200	8*1200

## 5 Conclusion

In this paper based on redesign of distribution system, changing of medium voltage level in order to have resilience network have been explained in a real and experimental context as Ahvaz grid. The proposed method had some main steps which clear many kind of challenges in upgrading proses and shown cases of initial unchanged and final renew system included power flow studies, lines loading and voltage criteria, equipment exchange, insulation requirement, short circuit and protection coordination, mechanical reinforcement poles. The results realized satisfied specifications of decreasing and increasing voltage level of two substation Ahvaz utility to 20 kV as expected in the papers sections. Also it has to say that the loading constraints such as maximum current capacity of lines, voltage index values and resizing of conductors, short circuit current change for selection of equipment and coordination of protecting relays are some of the challenges that investigated in this paper. economical investigation and energy pricing for future work and implementations can be suggested. According to case study input data, the Ahvaz distribution network consists of two sections, the west and the east, which have voltage levels of 33 and 11 kV. By reducing the voltage level from 33 kV to 20 kV, line current increase, and maybe thermal challenge will be shown in this change. Also voltage decreasing from 33 kV to 20 kV may be necessary to replace and uprating the existing conductor to new that has higher cross section and weight. And decreasing and increasing voltage from 33 kV to 11 kV–20 kV caused respectively increasing and decreasing of short circuit current in 1.65 and 0.55 times.

It has to be noticed that this proposal is associated with a passive electric distribution network although active elements as new experiment can be investigated for other case study but the main result which have been attended base on pick of load will not change such as line capacity or mechanical calculations.

## Data availability statement

In this paper the authors do not have permission to share data.

## CRediT authorship contribution statement

**Hadi Norouzi:** Writing – review & editing, Writing – original draft, Visualization, Supervision, Project administration, Methodology, Investigation, Conceptualization. **Mahdi Golchoob Firoozjaee:** Software. **Majid Rezaei:** Methodology, Investigation.

## Declaration of competing interest

The authors declare that they have no known competing financial interests or personal relationships that could have appeared to influence the work reported in this paper.
